# Advancements in research on the association between the biological CLOCK and type 2 diabetes

**DOI:** 10.3389/fendo.2024.1320605

**Published:** 2024-05-30

**Authors:** Hui Cheng, Dayuan Zhong, Yimei Tan, Menghe Huang, Sun Xijie, Hong Pan, Zixian Yang, Fangmei Huang, Feifan Li, Qizhi Tang

**Affiliations:** ^1^ Nanhai Hospital of Traditional Chinese Medicine, Jinan University, Foshan, China; ^2^ Institute of Traditional Chinese Medicine, Jinan University, Guangzhou, China; ^3^ Graduate school, Guangzhou University of Chinese Medicine, Foshan, China

**Keywords:** biological clock, type 2 diabetes, circadian rhythm, clock genes, lifestyle

## Abstract

Due to the Earth’s rotation, the natural environment exhibits a light-dark diurnal cycle close to 24 hours. To adapt to this energy intake pattern, organisms have developed a 24-hour rhythmic diurnal cycle over long periods, known as the circadian rhythm, or biological clock. With the gradual advancement of research on the biological clock, it has become increasingly evident that disruptions in the circadian rhythm are closely associated with the occurrence of type 2 diabetes (T2D). To further understand the progress of research on T2D and the biological clock, this paper reviews the correlation between the biological clock and glucose metabolism and analyzes its potential mechanisms. Based on this, we discuss the potential factors contributing to circadian rhythm disruption and their impact on the risk of developing T2D, aiming to explore new possible intervention measures for the prevention and treatment of T2D in the future. Under the light-dark circadian rhythm, in order to adapt to this change, the human body forms an internal biological clock involving a variety of genes, proteins and other molecules. The main mechanism is the transcription-translation feedback loop centered on the CLOCK/BMAL1 heterodimer. The expression of important circadian clock genes that constitute this loop can regulate T2DM-related blood glucose traits such as glucose uptake, fat metabolism, insulin secretion/glucagon secretion and sensitivity in various peripheral tissues and organs. In addition, sleep, light, and dietary factors under circadian rhythms also affect the occurrence of T2DM.

## Graphical Abstract

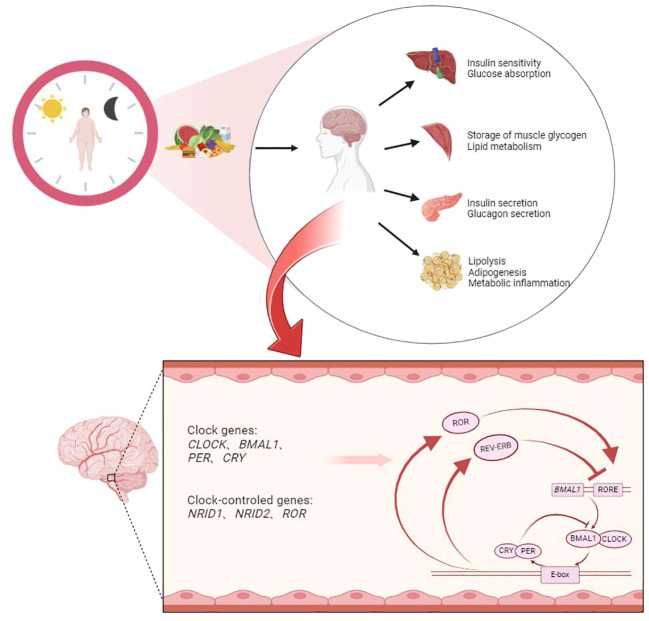

## 1 Introduction

Diabetes is a systemic metabolic disorder caused by various factors such as genetics, environment, and lifestyle (1). Diabetes includes four main types: Type 1 diabetes (T1D), Type 2 diabetes (T2D), Gestational diabetes (GD), and Other diabetes (OD) (1). Among them, T2D accounts for approximately 95% of all diabetes cases (2). T2D often leads to various complications, resulting in a decreased quality of life for patients and imposing a significant economic burden (3). The occurrence of T2D is mainly associated with impaired pancreatic β-cell function and insulin resistance (4). Existing research shows an accelerating trend in the prevalence of T2D in recent years (5). The high prevalence of T2D is likely a result of unhealthy lifestyles (6). Various physiological activities and habits in organisms exhibit clear rhythmic patterns, such as endocrine, sleep, and body temperature, all of which are regulated by the Biological Clock (BC), which governs the intrinsic rhythms of the entire organism (7). Poor lifestyle choices often lead to disturbances in the organism’s biological clock before the onset of T2D, suggesting a possible association between the biological clock and the development of T2D (8, 9). As research on the biological clock advances, its relationship with T2D is gradually being recognized. To further understand the progress of research on T2D and the biological clock, this paper collects and summarizes studies on the relationship between the circadian rhythm, autonomic nervous system, endocrine hormones, and T2D from multiple perspectives, aiming to elucidate the relationship between T2D and the biological clock and explore the possibility of preventing and treating T2D from the perspective of the biological clock.

## 2 Core mechanisms of the BC system

### 2.1 Composition and structure of the BC system

Due to the Earth’s rotation, the natural environment exhibits a light-dark diurnal cycle close to 24 hours. Sunlight serves as a crucial source of energy for organisms, and the light-dark diurnal cycle affects the energy intake of organisms. In order to adapt to this energy intake pattern, organisms have developed their own diurnal rhythms over extended periods. Research indicates that the BC, an endogenous autonomous internal system, is a significant source for generating and regulating rhythms in organisms (10), capable of driving various metabolic and related physiological behaviors. The associated intrinsic rhythms are interspersed across different time periods, blood pressure, body temperature, hormones, sleep-wake cycles, and various tissues of organisms, thus constituting the BC system with different hierarchical structures. Organisms can anticipate environmental changes in advance through BC and make adaptive adjustments in a timely manner. In the early 18th century, a French astronomer discovered the autonomous oscillation phenomenon of plants following the Earth’s rotation, thereby proposing the concept of BC (11). Subsequent validation experiments have confirmed the existence of BC (12). The BC system of organisms consists of Central BC and peripheral BC (13). Central BC serves as the ‘pacemaker’ of organismal rhythms, located in the suprachiasmatic nucleus (SCN) of the hypothalamus. For most mammals, it can be guided by external light signals (14). Its intrinsic mechanism involves the retina of the eyes receiving light signals and transmitting them to the SCN, which then establishes connections with other peripheral BC through various pathways. In addition to this, both can establish connections through the nervous system, temperature regulation, and humoral pathways (15–18). The peripheral BC system exists in organs such as the heart, liver, spleen, lungs, and kidneys, composed of non-SCN brain areas and peripheral tissue organs’ BC, driving the diurnal rhythm expression of specific genes and playing irreplaceable roles in corresponding tissues (19). In addition to receiving signals from the SCN, peripheral BC can also be reset by external signals (20). The coordinated action of central BC and peripheral BC together maintains the normal operation of organisms.

### 2.2 Molecular BC system

The regulatory mechanism of BC is formed by an autoregulatory transcription-translation feedback loop (TTFL). The core BC genes of this loop include brain and muscle aryl hydrocarbon receptor nuclear translocator-like 1 (*Bmal1*), circadian locomotor output cycles kaput (*CLOCK*), period homologs 1, 2, and 3 (*Per1/2/3*), cryptochrome homologs 1 and 2 (*Cry1/2*) (21).

The core mechanism of MBC involves the formation of a dimer of the transcriptional activators CLOCK and BMAL1 in the cytoplasm, which then binds to the upstream promoter containing E-box DNA regions to activate the transcription of *Cry1,Cry2,Per1,Per2*, and *Per3* (22). After CRYs and PERs proteins mature and form dimers and enter the nucleus, they in turn inhibit the transcriptional activity of CLOCK and BMAL1, thus presenting a negative feedback transcriptional regulatory loop oscillating with a period of approximately 24 hours (22). In addition to this main molecular feedback loop, the mechanism of MBC can also be regulated by branching pathways through Nuclear receptor subfamily 1 group D member 1, 2 (also known as NR1D1 and NR1D2) and RORα/β to regulate Bmal1 transcription (23). The specific regulatory pathway involves the competitive binding of homologous proteins NR1D1 (or homologous protein NR1D2) with ROR to the binding site on the ROR response element (RORE), and then jointly regulate the expression of *Bmal1* and *Cry1* genes. Many studies have shown that the regulation mechanism of *Bmal1* and *Cry1* genes is closely related to BC. Research has shown that the *Bmal1* gene affects the decrease in the transcription level of related genes and homologous proteins and the disappearance of circadian rhythms (24, 25). Knockout of the NR1D1 protein increases the transcription of BMAL1 at the low expression time point in brown adipose tissue, thereby reducing the amplitude of this transcription rhythm (26). At the same time, the loss of the *Bmal1* gene can also increase the transcription level of *Cry1* (27). In addition, these core clock genes also regulate the rhythmic expression of other genes that can affect complex physiological changes in the body.

The human *CLOCK* gene is located on the long arm of chromosome 4 (4q12), comprising 20 exons with a protein-coding sequence of 2538 bp. Due to the histone acetyltransferase activity of CLOCK protein, histone acetyltransferase can regulate chromatin remodeling, influencing the transcription and expression of rhythmic genes (28). The *CLOCK* gene is a core gene regulating the intracellular BC, and it was initially selected for cloning in mice, indicating its central role in mammalian molecular oscillations (28). Relevant experiments have shown that glucose tolerance abnormalities, reduced insulin secretion, and elevated blood glucose levels observed in mice are associated with *CLOCK* gene knockout, demonstrating that the *CLOCK* gene affects pancreatic β-cell secretion function and consequently influences blood glucose changes in organisms (29). Furthermore, related research has also demonstrated a close association between *CLOCK* gene single nucleotide polymorphisms (SNPs) or *CLOCK* gene deletion and T2D, obesity, and some metabolic diseases (30). Additionally, Marcheva et al. (31) found that mice with *CLOCK* gene knockout exhibited glucose tolerance abnormalities and reduced insulin secretion. Moreover, as mice age, defects in pancreatic islet cell size and proliferation emerge. Rabinovich-Nikitin et al. (32) found that loss of *CLOCK* gene activity impairs mitochondrial metabolic function, resulting in impaired mitochondrial autophagy, and oxidative stress caused by mitochondrial dysfunction promotes the occurrence of glucose metabolism abnormalities. Turek et al. (33), through animal experimental models, also demonstrated that *CLOCK* gene knockout mice not only exhibited severe disruption of daily feeding rhythms but also manifested overeating, obesity, hyperlipidemia, and hypoglycemia, accompanied by the development of metabolic diseases such as obesity and metabolic syndrome.

The *Bmal1* gene is an important transcription factor that regulates normal rhythms in organisms. The human *Bmal1* gene is mainly expressed in the brain, skeletal muscles, and heart, and it is upstream in the clock system. Its product, BMAL1 protein, is one of the factors involved in the positive feedback regulation of circadian rhythm feedback. Bmal1 protein mainly exists in non-brain fat tissues, and both BMAL1 and CLOCK proteins are involved to some extent in glucose metabolism processes (34). Regarding the *Bmal1* gene, related studies have demonstrated that specific knockout of the *Bmal1* gene inhibits insulin secretion, leading to glucose intolerance (35). Loss of *Bmal1* causes upregulation of uncoupling protein 2 on mitochondria, ultimately leading to mitochondrial uncoupling, which, through a series of processes, eventually results in decreased insulin secretion (36). Additionally, it has been mentioned that Bmal1 protein regulates insulin expression by binding to the E-box on the promoter and promotes exocytosis of insulin secretory vesicles (37). Loss of *Bmal1* is associated with pancreatic β-cell failure-induced type 2 diabetes. By studying the consequences of Bmal1 inhibition in insulinoma cell lines (INS-1), it was found that *Bmal1* knockout impairs glucose-stimulated β-cell insulin secretion and causes adverse changes in mitochondrial membrane potential and mitochondrial structure. Finally, it is considered that the loss of this gene may impair pancreatic β-cell function through the mitochondrial signaling pathway in INS-1 cells (38). Mitochondrial dysfunction can impair glucose utilization and insulin secretion in β-cells (39, 40).

**Figure 1:**
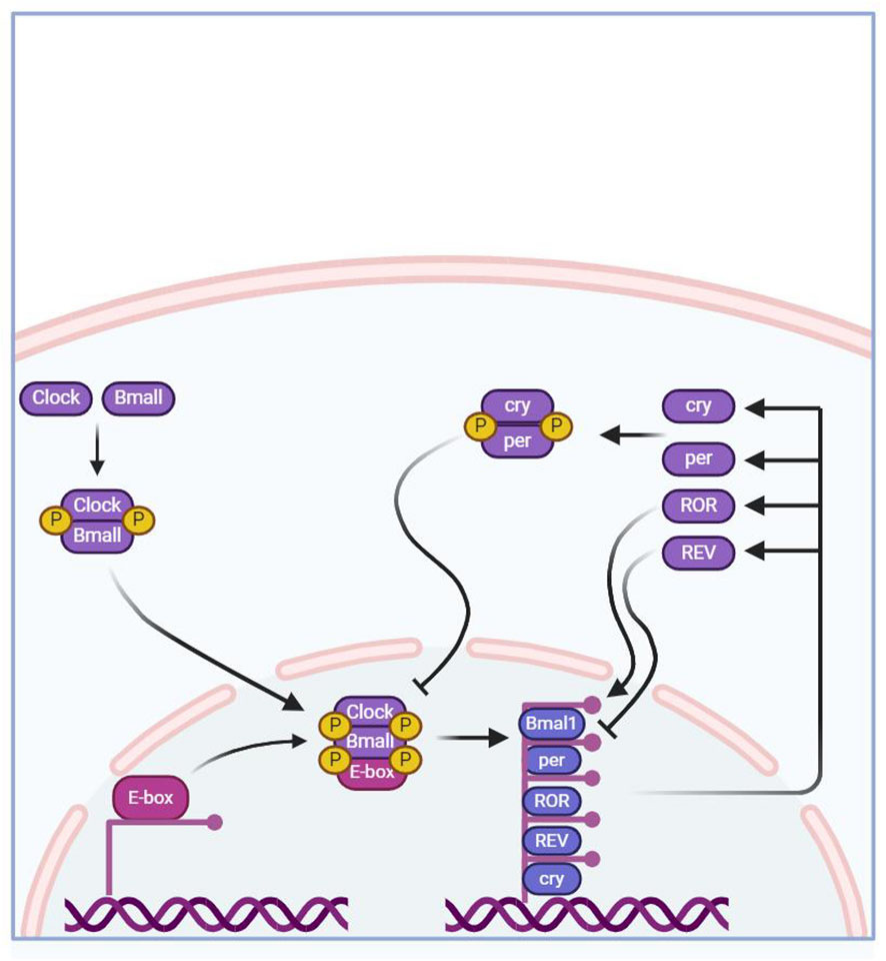
The core regulation mechanism of MBC.

Related to the *Per1/2/3* genes, studies have shown that deletion of the *Per2* gene in mice leads to elevated insulin levels in the blood (35). Compared with wild-type mice, gene deletion ultimately leads to a decrease in insulin clearance rate in mice, which is not conducive to gluconeogenesis (35). Similarly, the expression of *Cry1* and *Cry2* genes blocks the phosphorylation of cAMP response element-binding protein and inhibits glucagon-mediated cAMP increase, which mainly leads to the expression of fasting gluconeogenesis-related genes. Conversely, if Cry genes are deleted, the inhibitory effect on hepatic gluconeogenesis is relieved (41). In addition, disruption of the negative regulatory factors PERs and CRYs of the clock system is associated with hyperinsulinemia (42). The *Per2* gene plays a role in mediating glucose homeostasis. Related studies have found that Per2-deficient mice enhance glucose-stimulated insulin secretion, impair insulin-degrading enzyme, and increase plasma insulin levels (43). Tao et al. inhibited the expression of the gene *per* by selecting model animals with only one *Per* gene and found a decrease in the products of the glycolysis process, indicating that lowering the expression of the *Per* gene inhibits the glycolysis pathway (44), confirming that deletion or loss of function of *Per1/2/3* genes leads to lipid metabolism disorders. The detailed mechanism as shown in **Figure 1**.

## 3 BC system and its relevance to T2D

### 3.1 Central BC system and the association with diabetes mellitus

To confirm the relationship between central BC genes and the onset of diabetes mellitus, researchers selectively disrupted several critical regions of the hypothalamus in rats to interfere with their circadian activity rhythms. Subsequently, they verified the relationship between these lesions and circadian rhythm disruption through a series of microscopic examinations of brain slices. The final results indicated that damage to the hypothalamus was associated with the disappearance of diurnal metabolic activities and behaviors in the organism, thereby demonstrating the crucial importance of the SCN in BC generation (45).

**Figure 2:**
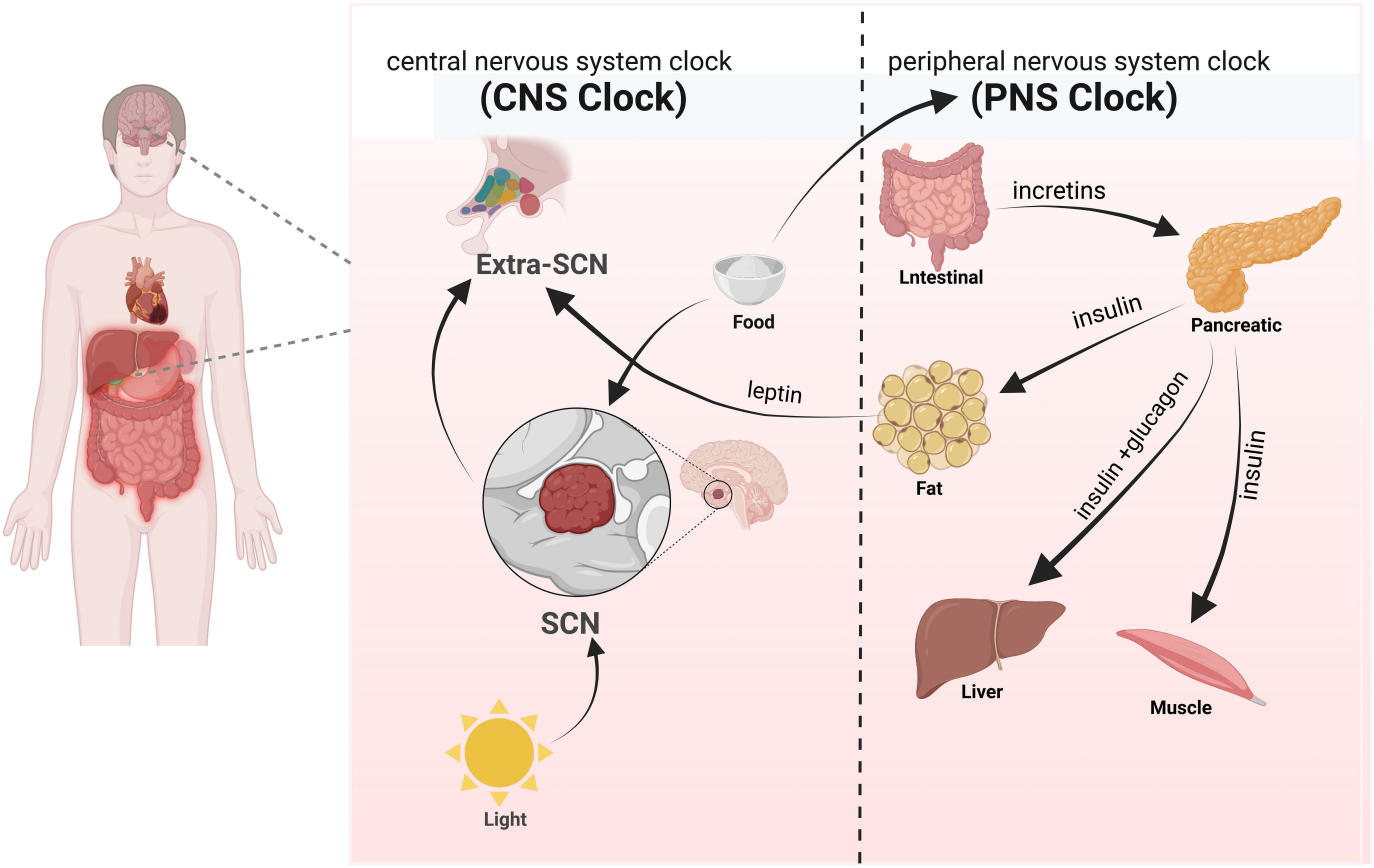
BC system (central and peripheral) involved in metabolic processes.

Brain and interorgan signaling among the liver, muscles, gastrointestinal tract, and adipose tissue collectively participate in regulating organismal metabolism (46) (**Figure 2**). Signals from light and food stimuli received by the brain trigger the expression of central BC system genes, which are then relayed by the central nervous system through hormone secretion and neural signals to regulate calorie intake and behavior, thereby influencing metabolic processes in peripheral tissues. The retina receives light stimuli and transmits information to the SCN in the brain, driving diurnal rest and activity rhythms and determining feeding-fasting cycles. The rhythmic metabolic signals induced by scheduled food intake synchronize the body’s internal clock with the external environment (47). The SCN transmits light stimuli information to all clocks within the body through various pathways to synchronize the timing and amplitude of diurnal rhythms in all organs and tissues (48). The interplay between different hormones in the peripheral BC system regulates feeding behavior and glucose homeostasis in response to food stimuli. Nutrients in food can stimulate various nutrient-sensing receptors on enteroendocrine cells in the small intestine, promoting the secretion of intestinal hormones such as incretins, which play important roles in adjusting maladaptive eating habits, enhancing insulin secretion, and increasing insulin sensitivity (49, 50). Pancreatic tissues release insulin, glucagon, and other related hormones to maintain blood glucose stability. Simultaneously, adipose tissue secretes leptin, a satiety signal that is proportional to overall body fat mass and is associated with circadian rhythms (51, 52). It acts on other regions (Extra-SCN) controlling neurons to establish connections with the SCN, jointly regulating various metabolic processes within the body. The human clock gene system plays a significant regulatory role in maintaining diurnal variations in glucose metabolism (53).

### 3.2 Peripheral BC system and its relevance to diabetes mellitus

#### 3.2.1 Liver BC genes and the onset of diabetes mellitus

The liver, as the primary metabolic hub in the body, stores glucose in the form of glycogen. The body’s BC, as the basis for balancing hepatic glycogen synthesis and breakdown, is associated with relevant genes including the D-box binding protein (DBP), *Cry1*, and *Per2* (54). Studies have found that these transcription factors not only directly regulate cell BC output genes like DBP but also modulate other BC output genes and core clock genes (55). Additionally, they can influence many genes containing E-boxes by antagonizing the activity of *CLOCK* and *Bmal1* genes, ultimately affecting and regulating the timing and amplitude of diurnal BC rhythms (55). The *CRY1* gene can regulate glucose metabolism by affecting glucocorticoid receptor and modulating the signals of enzymes related to glucagon (54). *PER2* and *CRY1* are a pair of negative regulatory BC genes that play indispensable roles in maintaining the stability of diurnal rhythms (55). It has been found that Per2-deficient mice and Cry1-deficient mice exhibit glucose intolerance (54). Excessive expression of *Cry1* increases insulin sensitivity, and its binding with the small molecule KL001 alters the cycle of BC, leading to its prolongation and inhibiting glucagon-induced gluconeogenesis (56). In summary, disruption of BC rhythms leads to increased or decreased body weight, elevated blood glucose levels, impaired glucose tolerance, and diminished insulin secretion in normal mice. The expression of liver BC genes Dbp, *Per2*, and *Cry1* is downregulated, resulting in increased body weight and decreased blood glucose levels in T2D mice (57). It is evident that the normal expression of liver *CLOCK* and *Bmal1* genes plays a crucial role in regulating hepatic glycogen synthesis and liver insulin sensitivity. Therefore, when BC genes in the liver are abnormally expressed, the disruption of glucose metabolism leads to the occurrence of T2D.

#### 3.2.2 Muscle BC genes

The skeletal muscle, one of the largest organs in the human body, accounts for about 45% of total body mass. More than 2300 genes are expressed in a diurnal rhythm pattern in skeletal muscle, participating in various processes including muscle formation, transcription, and metabolism (58). The diurnal rhythm of skeletal muscle may indirectly transmit through light or directly through feeding and activity times to the SCN (59). Skeletal muscle molecular clocks not only need to adapt to the environment but also need to synchronize with rhythms in other tissues, which is crucial. When diurnal rhythms are disrupted, changes related to fiber type, muscle structure, decreased mitochondrial respiratory function, and impaired muscle function occur (60). In addition to these, adverse effects on metabolic health include impaired glucose tolerance and diminished insulin sensitivity (61). Glycogen is the storage form of glucose in muscles, and normal BC in the body is a prerequisite for balancing glycogen synthesis and breakdown in muscles. The first paper defining diurnal rhythm gene expression in skeletal muscle was published in 2007, identifying 215 diurnal rhythm genes (62). In human skeletal muscle, the *FBN1* gene is present in mesenchymal stem cells, osteoblast-like cells, and mesenchymal stem cells, with the C-terminal region of this gene containing Asprosin (63). Studies have shown that it acts as a central appetite stimulant or fasting-induced glycogen protein hormone, and its gene expression is also influenced by circadian rhythms, primarily released into the bloodstream from white adipose tissue (63). This hormone plays a crucial role in regulating metabolic disorders such as diabetes and obesity (64). Its main mechanism in the formation of T2D is through promoting gluconeogenesis, impairing the secretion function of pancreatic β-cells, and causing insulin resistance. Romere et al. (65) confirmed through *in vivo* and *in vitro* experiments that Asprosin can directly act on liver cells, promoting gluconeogenesis through the cyclic adenosine monophosphate (cAMP) - protein kinase A (PKA) signaling pathway. Other studies have found that this hormone exacerbates inflammation by activating the Toll-like receptor 4 (TLR4) - c-Jun N-terminal kinase (JNK) pathway, ultimately leading to apoptosis of pancreatic β-cells (66).

#### 3.2.3 Pancreatic BC genes and diabetes relationship

The pancreas, consisting mainly of pancreatic α-cells and pancreatic β-cells and other related cells, is the endocrine part of the pancreas. Pancreatic β-cells secrete insulin to lower blood sugar, while pancreatic α-cells secrete glucagon to raise blood sugar. T2D is mainly due to pancreatic β-cell damage or insulin resistance, indicating a close relationship between the pancreas and T2D. Studies have shown that by constructing a mouse sleep rhythm disorder model using the classic water platform method, aiming to explore the effect of sleep rhythm disorder on glucose metabolism and pancreatic function in normal and diabetic mice, it was found that interfering with the normal sleep rhythm of mice in the control experiment resulted in increased insulin secretion in diabetic mice compared to the control group. This indicates that insulin secretion is closely related to the BC rhythm and is one of the key factors in maintaining the physiological homeostasis of blood sugar (67). Peng Xiaoyong et al. quantitatively evaluated the differences in liver and pancreatic fat infiltration content and distribution between diabetic patients and ordinary healthy individuals using IDEAL-IQ technology (3D asymmetric echo’s least squares estimation algorithm iterative water and fat separation technology). The results showed that liver and pancreatic fat deposition are closely related to the occurrence and development of T2D (68). Inconsistent findings show that humans display significantly increased glucose and insulin levels after regular meals, with some subjects reaching pre-diabetic levels (69), indicating that circadian rhythm disruption leads to decreased insulin sensitivity (increased demand for insulin secretion) and compensatory β-cell response to elevated glucose levels (insufficient insulin to lower blood sugar) (70).

#### 3.2.4 Fat BC genes and diabetes relationship

Fat is not uniformly distributed in the body; its tissue grows and develops in different parts of the body and varies among different species of animals (71). In mammals, adipose tissue can be classified into visceral adipose tissue (VAT) and subcutaneous adipose tissue (SAT) and ectopic adipose tissue (EAT) according to its distribution, and according to morphology, function, and developmental origin, it can be divided into white adipose tissue (WAT) and brown adipose tissue (BAT) (72). Adipose tissue not only stores energy but also has endocrine functions (73).

Insulin can promote the synthesis and storage of fat and inhibit its breakdown. It promotes glucose uptake and triglyceride synthesis by acting on fat tissue, while inhibiting TG hydrolysis and the release of free fatty acids (FFAs), thus lowering blood sugar. Generally, insulin effectively inhibits fat breakdown in the body, but this inhibitory effect is weakened in patients with T2D (74). In such individuals with metabolic disorders, fat mobilization leads to the generation of large amounts of FFAs, which are transported to other tissues, exacerbating fat deposition and insulin resistance in other organs (75). The increase in FFA concentration plays a crucial role in the pathogenesis of insulin resistance, as confirmed by numerous previous studies (76). Compared to subcutaneous adipose tissue, visceral adipose tissue has relatively lower insulin sensitivity, making it more prone to release large amounts of FFAs, affecting the normal metabolism of insulin, reducing the sensitivity of other tissue organs to insulin, and thereby causing elevated blood sugar (77).

The process of regulating insulin secretion by adipose tissue is influenced by the endogenous biological clock (78). Researchers have developed an *in vitro* model of circadian biology in human fat using differentiated adipocytes derived from stem cells and found that there is a circadian clock regulated in fat cells (79). Light stimulation affects the formation and breakdown of tissue cells themselves, thereby affecting their actions with other cell factors or target organs (80). Furthermore, some cell factors produced during the breakdown or generation process of fat cells also exhibit circadian rhythm (81). Under continuous light conditions, adipose tissue further affects skeletal muscle insulin sensitivity via exosomes (82). Studies have also confirmed that exosomes are associated with sleep time in the biological clock, with increased exosome secretion from fat cells observed in rotating shift workers, further suggesting that exosomes may be a bridge between the biological clock and insulin sensitivity (82). The disruption of adipocyte circadian rhythms also regulates the expression of related inflammatory factors, leading to inflammation in related tissues and promoting the development of T2D (83, 84). The main components of the molecular clock directly participate in regulating inflammatory responses, including key regulatory factors such as *Bmal1, CLOCK, Rev-erbα, and Per/Cry* (85). Fibrosis and neovascularization were significant features found in the fat depot of rotating shift workers (86). The crown-like structures composed of abundant macrophages around necrotic fat tissue are indicative of significant macrophage infiltration, a hallmark of fat inflammation (87). Moreover, Xuekai Xiong et al. found that chronic circadian disruption results in significant adipose tissue enlargement, accompanied by pronounced inflammatory responses and fibrosis sequelae, which easily lead to insulin resistance and ultimately promote the occurrence of T2D (88). With further discoveries regarding the adipose tissue biological clock, there are more intrinsic mechanisms to be explored. As a key target organ of insulin action, adipose tissue plays an important role in the metabolism of glucose and lipids in the body.

### 3.3 Influence of circadian rhythms on blood glucose control in diabetes patients

#### 3.3.1 Circadian rhythms and glucose metabolism

BC is involved in regulating various metabolic processes, and circadian rhythms under the BC system play an important regulatory role in glucose metabolism (89). In terms of glucose metabolism, normal circadian rhythms play a crucial role in stabilizing internal glucose metabolism. Researchers have used morning-evening type measures (different sleep type tables) to statistically analyze data to determine the circadian rhythm types of test subjects. The lower the score, the more inclined the type is toward eveningness (90). Studies have found that individuals with an eveningness preference have poorer blood glucose control, indicating a close relationship between blood glucose control and circadian rhythms (91). Insulin secretion in T2D patients exhibits a circadian rhythm opposite to blood glucose fluctuations, peaking at night (19:00) and gradually decreasing overnight, reaching its lowest point in the morning (08:00) (92). This phenomenon is believed to contribute to the occurrence of the “dawn phenomenon” in diabetes patients (26). Furthermore, studies have indicated alterations in the number of SCN neurons in T2D patients, characterized primarily by a reduction in the relevant neuronal population, which may impact the rhythmicity of central BC (93). Numerous experimental studies have consistently demonstrated a significant association between the development of diabetes and intrinsic disruptions in the organism’s glucose metabolism rhythm. Disrupted circadian rhythms in animal diabetes models have been shown to accelerate the loss of pancreatic β-cell function, thereby adversely affecting insulin secretion (94). Additionally, research has found that disruptions in the circadian rhythm system leading to impaired pancreatic function within the organism, as well as dysfunction in pancreatic BC, may ultimately increase the likelihood of T2D onset. This also elucidates the potential mechanism by which common circadian rhythm disruptions in modern life can lead to pancreatic failure in T2D patients (95). Liu Mengdi and colleagues conducted a study to investigate the effects of circadian rhythm disruption on insulin secretion levels. They observed changes in body weight, blood glucose, oral glucose tolerance test (OGTT), and insulin tolerance test (ITT) blood glucose curve areas under the curve (AUC) in different groups of mice (normal control group, T2D + normal circadian rhythm group, normal + circadian rhythm disruption group, T2D + circadian rhythm disruption group, with 12 mice in each group), and detected mouse liver clock genes using fluorescence quantitative PCR (qRT-PCR). The results showed that mice in the circadian rhythm disruption group exhibited significant increases in blood glucose levels, indicating a certain influence of circadian rhythm disruption on the body weight, blood glucose, and insulin secretion levels of both normal and T2D mice (57).

The circadian rhythm and the onset of T2D primarily manifest in different stages of sleep duration; several days of complete or partial sleep restriction can lead to decreased glucose tolerance and impaired insulin sensitivity, resulting in elevated blood sugar levels. Some large-scale data analyses have shown (96) that the risk of developing T2D has a U-shaped relationship with sleep duration, ultimately considering 7–8 hours of sleep duration to be optimal (96). This “U-shaped relationship” also indicates that both excessive and insufficient sleep durations increase the risk of diabetes to a certain extent (96). Previous research has found that normal individuals with either prolonged (sleep duration > 8 hours) or shortened (sleep duration < 5 hours) sleep durations significantly increase fasting blood glucose and glycated hemoglobin levels (96).

Related studies suggest that controlling daily eating times can control blood sugar levels in the body, with food intake timed during the daytime to align with internal rhythms, thereby better controlling blood sugar and weight. However, this conclusion requires further consideration (97). Other studies have found that the body’s metabolism reaches its peak in the early morning or late afternoon physiologically, suggesting that it is more appropriate to eat during the daytime than at night (26). Research exploring the connection between daily metabolic rhythms and circadian rhythms has found that some rhythms peak physiologically in the early morning or late afternoon (26). Additionally, studies have found that the thermic effect of food is higher in the morning than in the evening, implying that early daytime is the optimal time for food intake (98). Eating at night disrupts the body’s metabolic rhythm, affecting physiological functions such as insulin secretion and glucose absorption, thereby inducing the onset of T2D (98).

In addition to eating times being linked to internal metabolism, light sources also influence the body’s metabolic processes to some extent. An increase in artificial light at night leads to circadian rhythm disruption within the body, ultimately increasing the risk of T2D (99).

The circadian rhythm of blood sugar metabolism is closely related to the circadian rhythm controlled by BC. Disruption of circadian rhythms can affect the circadian rhythm of blood sugar metabolism, thereby altering the blood sugar levels of diabetes patients (100). Represented by shift workers, many studies on circadian rhythm disruption focus on shift workers. Relevant research shows that compared to normal daytime workers, shift workers have a higher risk of developing T2D, with their risk being approximately 40% higher. This also indicates a proportional relationship between the incidence of diabetes and shift times (101). Bass et al. also confirmed through studies that the length of shift work is related to the incidence of T2D, with shift workers having a higher incidence of T2D than regular workers (102). In the United States, VETTER C et al. observed and monitored nurses’ daily work routines and conducted health tests, finding that shift workers had a 40% increased risk of T2D. These studies collectively demonstrate a positive correlation between the frequency and duration of shift work and the risk of developing T2D (103). Shift work exacerbates the likelihood of blood sugar disruptions in diabetic patients, thereby increasing the likelihood of developing related metabolic diseases (104). Through assessments using sleep, mood, and diet questionnaires and subsequent testing of HbA1c levels, research found that HbA1c levels in shift workers were higher than in normal shift workers, confirming the association between disrupted circadian rhythms and poor blood sugar control in T2D patients (104). Understanding the fact that disrupted circadian rhythms can lead to disruptions in blood sugar metabolism is key to treatment. Therefore, reducing the occurrence of unhealthy circadian rhythm habits helps stabilize blood sugar control and reduces the incidence of T2D (104).

The disruption of BC rhythms is correlated with T2D across different age groups (105). Regardless of the population, the BC located in the SCN is somewhat impaired. For elderly individuals, as humans age, the volume and number of cells in the SCN in the brain gradually decrease, leading to degeneration of SCN neurons and weakening of the circadian rhythm (106). This also explains the widespread severe sleep problems and disruption of hormonal circadian rhythms found in the elderly (107–109). Similarly, disruptions in BC rhythms have been observed in young T2D patients (105), possibly due to modern lifestyle habits among young people leading to BC metabolic disruptions, resulting in the loss of SCN rhythmicity and disruption of normal BC rhythms, which may lead to the occurrence of metabolic syndrome and T2D. In summary, although the causes of BC disruptions in young and elderly individuals are unclear, the outcomes are consistent (105). The aforementioned data and research are sufficient to demonstrate the close connection between glucose metabolism, which is closely related to diabetes, and circadian rhythms.

#### 3.3.2 Impact of lifestyle circadian rhythm disruption on T2D

The etiology of T2D is complex, involving both congenital genetic factors and acquired environmental factors, including other uncontrollable factors (such as age, genetics, etc.) and controllable factors (such as diet, physical activity, etc.). Lifestyle under circadian rhythm is currently recognized as an important factor influencing the occurrence of T2D. Besides pharmacological treatments, lifestyle interventions can effectively reduce the incidence of T2D in high-risk populations (110). Adhering to a reasonable lifestyle according to circadian rhythm can effectively prevent and control the occurrence of diabetes. Here, three key factors (sleep rhythm, light rhythm, dietary rhythm) and their relationship with T2D are discussed.

##### 3.3.2.1 Sleep rhythm and T2D

Increasing research data show that quality sleep under normal circadian rhythm plays an irreplaceable role in glucose metabolism in the body. Therefore, maintaining a healthy and reasonable sleep schedule is essential for preventing T2D. As mentioned earlier, the duration of shift work is directly proportional to the incidence of diabetes, and individuals who sleep 7–8 hours per night have the lowest risk of developing diabetes (111). In addition to sleep duration, there are differences in the risk of disease between men and women, with men having a higher risk than women (112). Rutters et al. found that both excessive and insufficient sleep duration in men compared to those who sleep a healthy duration each night are associated with impaired glucose metabolism, leading to elevated blood sugar and increased risk of diabetes (112). For women, sleep duration may not significantly increase the risk of T2D. Over the past 50 years, the average sleep duration in humans has decreased by approximately 1.5–2 hours. Comparing the recent doubling of the diabetes incidence rate with that 50 years ago, it is undeniable that a normal sleep rhythm plays a crucial role in the prevention and treatment of T2D (113). There are intrinsic mechanisms within the body that exacerbate the occurrence of diseases in individuals with sleep disorders. Research has found that sleep disturbances can enhance insulin resistance, disrupt insulin β-cell function, and ultimately lead to disease occurrence through the induction of hypoxemia, inflammation, activation of the sympathetic nervous system, and hypothalamic-pituitary-adrenal axis (114).

Circadian rhythm is closely related to human physical and mental health. Overall, human sleep types can be classified into morning types, evening types, and intermediate types (90). Morning types refer to individuals who prefer to sleep and wake up early and are more efficient in completing learning and work tasks in the morning than in the afternoon or evening. Evening types, on the other hand, prefer to sleep and wake up late, and their efficiency in learning and work at night is significantly higher than during the day. The intermediate type has no specific preference and is the largest proportion. Although a large number of studies have indicated that morning-type sleep is a factor in maintaining healthy and positive psychology, evening-type sleep poses a significant threat to health (90). However, an interaction analysis between sleep type and shift work found that among night shift workers, compared to morning types, evening types have a lower risk of developing T2D (115). However, compared to intermediate types, morning types are at a higher risk of developing T2D with the prolongation of shift work hours (116). The above data illustrate that the occurrence of T2D is influenced by multiple factors, not only gender differences but also differences in the risk of T2D among individuals. Different types of sleep and lifestyle rhythms jointly affect the occurrence of metabolic diseases, with evening types possibly better coping with circadian rhythm disruption caused by night shift work, thereby mitigating the adverse effects of night shift work on diabetes.

##### 3.3.2.2 Light rhythm and T2D

The relationship between light and T2D mainly manifests in the involvement of the BC system in metabolic processes. After being stimulated by light, the retina transmits signals to the central SCN, which in turn transmits them to the peripheral BC system to regulate various tissues and organs of the body (117). The peripheral BC signal then acts back on the SCN through negative feedback regulation, maintaining consistency between the central BC and peripheral BC rhythms with the external environment. Through a series of complex processes in the body, the BC process is driven (117).

Endocrine metabolism in the body varies with light rhythm changes. During the day, the liver enhances glycogen synthesis, and pancreatic insulin secretion increases, thereby increasing insulin sensitivity (94). In the evening, the liver’s gluconeogenesis is enhanced, glycogen is broken down, pancreatic secretion of glucagon increases, and insulin sensitivity decreases, making it easier for blood sugar to rise after nighttime eating. The pineal gland in the brain secretes melatonin. Photoreceptive retinal ganglion cells in the eyes are stimulated by light, affecting the synthesis and release of this neurotransmitter, so this hormone exhibits various circadian rhythms including sleep (118). Melatonin is secreted at night in the circadian cycle, with its peak secretion occurring between midnight and 4 a.m. Therefore, the hormone’s level is lower during the day and higher at night (119). Research has found that melatonin is associated with the risk of T2D, and its action is mainly mediated by receptors MT1 and MT2, which participate in regulating insulin secretion in β-cells and glucagon secretion in α-cells (120). These two receptors are also expressed in the pancreas. Melatonin release stimulates pancreatic α-cells and promotes glucagon release. Melatonin secretion is influenced by light, affecting the secretion of insulin, glucagon, and somatostatin inside and outside the body (121).

For T2D patients, increased exposure to nighttime light (LAN) can elevate fasting and postprandial blood glucose levels, possibly due to reduced glucose uptake caused by intense light exposure (8). Light serves as a crucial mediator, synchronizing our body’s internal biological clock with the 24-hour day-night rhythm changes in the environment (47). Individuals in contemporary society may be exposed to artificial light available throughout the day, and this LAN exposure can lead to elevated postprandial glucose and insulin levels (122). Furthermore, studies have found that compared to dim light, bright light can influence glucose metabolism in the body at different times of the day (123). Clinical trials have also confirmed that combining exposure to bright light during the day with exposure to dim light at night can achieve an effective metabolic regimen (124).

##### 3.3.2.3 Dietary rhythms and T2D

The relationship between dietary patterns and T2D primarily manifests in excessive food intake and inappropriate meal timing. The former is mainly associated with overeating leading to overweight or obesity, closely linked to the occurrence of T2D (8). Obesity is a concurrent and leading factor in type 2 diabetes, and a high-fat, low-carbohydrate ketogenic diet (with carbohydrate content as low as 10%) has been found to result in weight loss, improvement in body composition indicators, reduction in blood glucose levels, and enhancement of cardiopulmonary function in overweight and obese patients with type 2 diabetes (125).

Appropriate meal timing is also crucial in preventing the occurrence of T2D, as a normal eating-fasting cycle plays a significant role in maintaining metabolic homeostasis under the control of molecular BC (126). Time-restricted feeding (TRF), a form of intermittent fasting that restricts eating to a few hours of animal activity, has been shown to improve circadian rhythms (127), maintaining normal circadian rhythms, with a close relationship between the two. Chaix et al. found that feeding animals the same diet under TRF (restricting food intake to 10 hours at night) compared to providing ad libitum food did not result in increased weight gain or related metabolic disorders (126). Experimental results indicate that BC maintains metabolic homeostasis by regulating daily rhythms of eating and fasting and balancing nutrient and cellular stress responses. Studies have also confirmed the potential of TRF in preventing the onset of metabolic disorders (126). Mice with BC defects under TRF can avoid glucose intolerance and insulin resistance, to some extent preventing the occurrence of T2D (126). In addition, diabetic patients have also been found to have their biological clocks destroyed. As highlighted in the manuscript: “Studies have shown abnormalities in the serum circadian-related genes CLOCK and some inflammatory factors in patients with T2DM, and there is a correlation between them (128). The body of T2DM patients is affected by abnormal blood sugar levels, including long-term damage to the intestinal mucosal barrier, leading to metabolic substances and bacterial translocation, thereby inducing abnormal immune dysfunction in the body, which positively damages related circadian genes (129). At the same time, many adverse lifestyle habits or practices can disrupt the expression of circadian genes, thereby altering the body’s circadian rhythms and causing some diseases, including T2DM (128).

These studies demonstrate the significant role of controlling meal timing, limiting nighttime eating, and regulating food intake in preventing the occurrence of T2D. Combining dietary therapy with insulin therapy in elderly T2D patients yields better therapeutic effects, effectively controlling blood sugar levels and reducing the risk of complications (130, 131).

## 4 Conclusion

In summary, this article establishes the correlation between the core mechanism of the BC system, understanding of central and peripheral BC systems, circadian rhythms, and the disruption of lifestyle circadian rhythms with T2D. The above research findings are sufficient to demonstrate that glucose metabolism can be influenced by the circadian rhythm system under the BC system, indicating that the body’s blood sugar fluctuations exhibit circadian rhythm. Therefore, disruption of circadian rhythms can have adverse effects on blood sugar control. In this context, appropriately adjusting dietary habits, improving sleep patterns, and regulating the expression of related BC genes can provide better treatment and prevention strategies for T2D. Although the relationship between the BC system genes and glucose metabolism is gradually becoming clearer, there is still much to explore in the future, such as how various genes under the BC system interact to cooperatively regulate insulin synthesis and secretion, and whether an intrinsic factor can be found to balance BC in T2D patients, thereby improving the metabolic disorders in the body. These aspects require further in-depth research.

## Author contributions

HC: Conceptualization, Visualization, Writing – original draft, Writing – review & editing. DZ: Conceptualization, Visualization, Writing – original draft, Writing – review & editing. YT: Conceptualization, Writing – review & editing. MH: Supervision, Writing – review & editing. SX: Conceptualization, Supervision, Writing – review & editing. HP: Conceptualization, Supervision, Writing – review & editing. ZY: Conceptualization, Writing – review & editing. FH: Conceptualization, Writing – review & editing. FL: Conceptualization, Writing – review & editing. QT: Conceptualization, Methodology, Writing – review & editing.
